# Supplementary material for: Feeding climate and biodiversity goals with novel plant-based meat and milk alternatives
Source: Nat Commun. 2023 Sep 12;14:5316. doi: 10.1038/s41467-023-40899-2 (PMC10497520; doi:10.1038/s41467-023-40899-2)
Supplement: Supplementary file 1 — Supplementary Information [file 41467_2023_40899_MOESM1_ESM.pdf]

Supplementary materials

Supplementary notes 1 Regional differences in demand for ASF

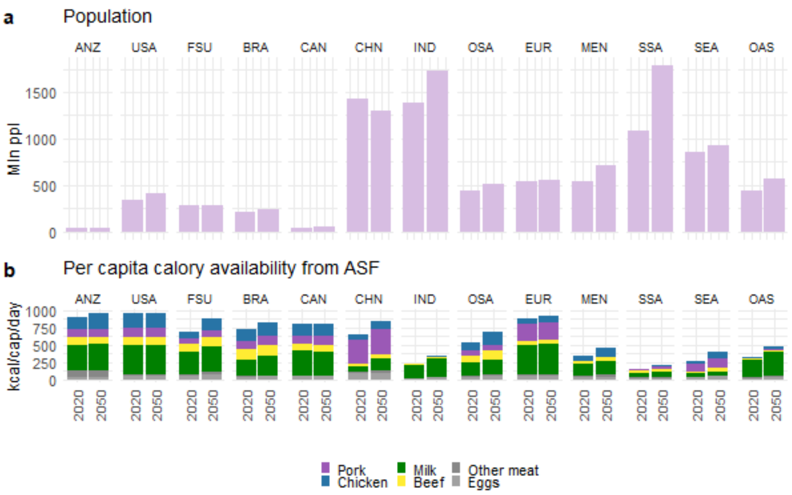

Figure S1 Population size and ASF consumption regionally in 2020 and 2050 in REF scenario

Regional abbreviations: Brazil (BRA), China (CHN), Former Soviet Union (FSU), India (IND), Middle East and North Africa (MEN), Other Asia (OAS), Other South America (OSA), Southeast Asia (SEA), Sub-Saharan Africa (SSA), Oceania (ANZ), Canada (CAN), Europe (EUR), United States (USA).

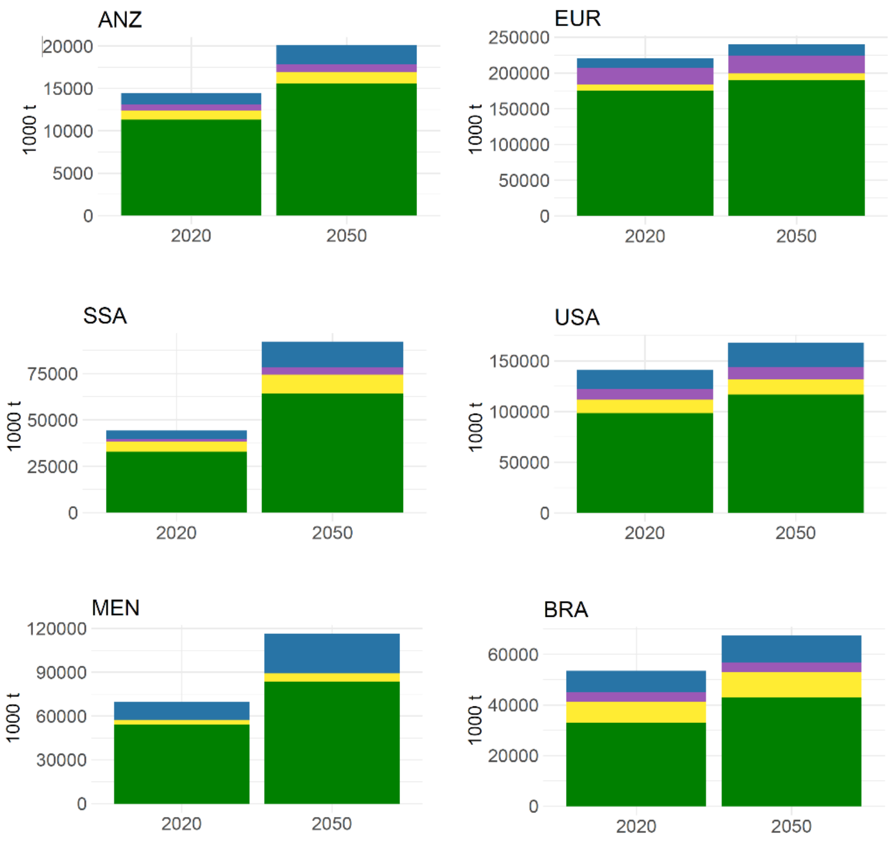

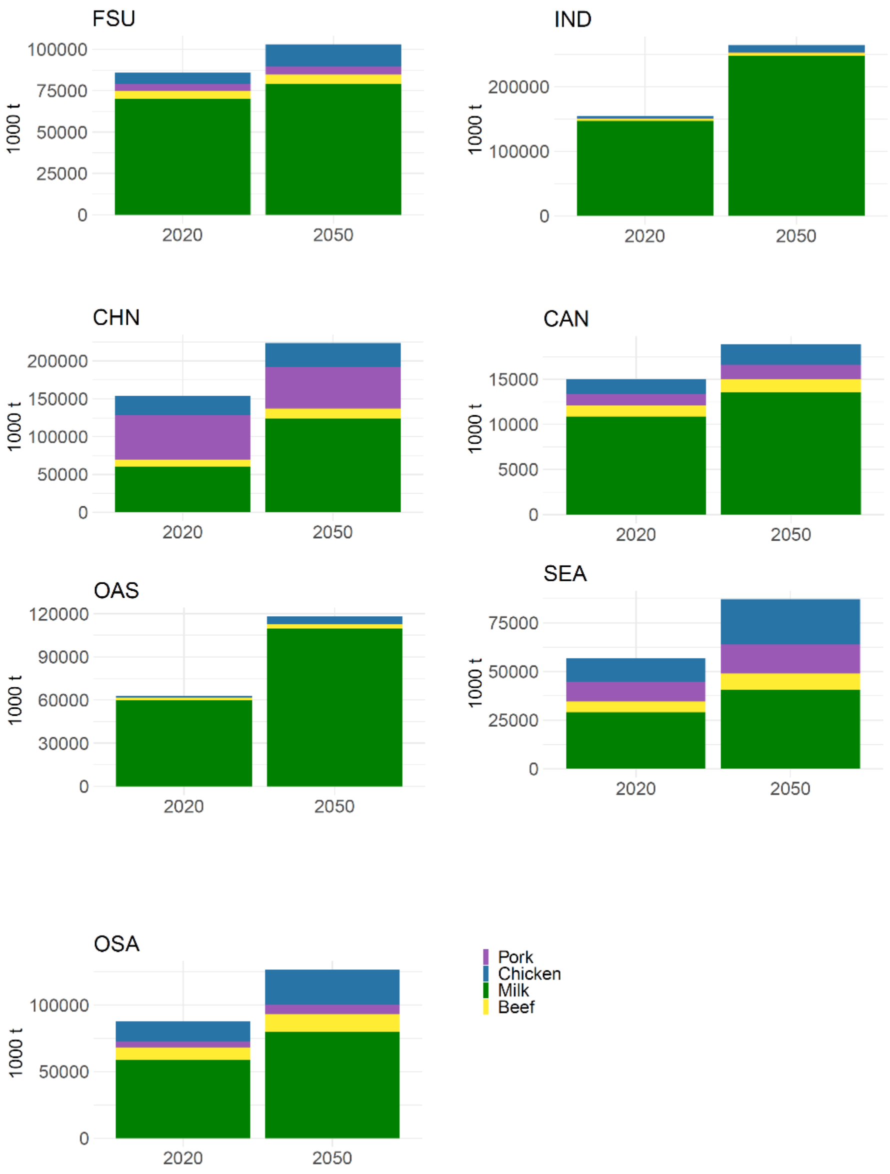

Figure S2 Regional per capita calory availability from the main ASFs in 2020 and 2050 REF

Regional abbreviations: Brazil (BRA), China (CHN), Former Soviet Union (FSU), India (IND), Middle East and North Africa (MEN), Other Asia (OAS), Other South America (OSA), Southeast Asia (SEA), Sub-Saharan Africa (SSA), Oceania (ANZ), Canada (CAN), Europe (EUR), United States (USA).

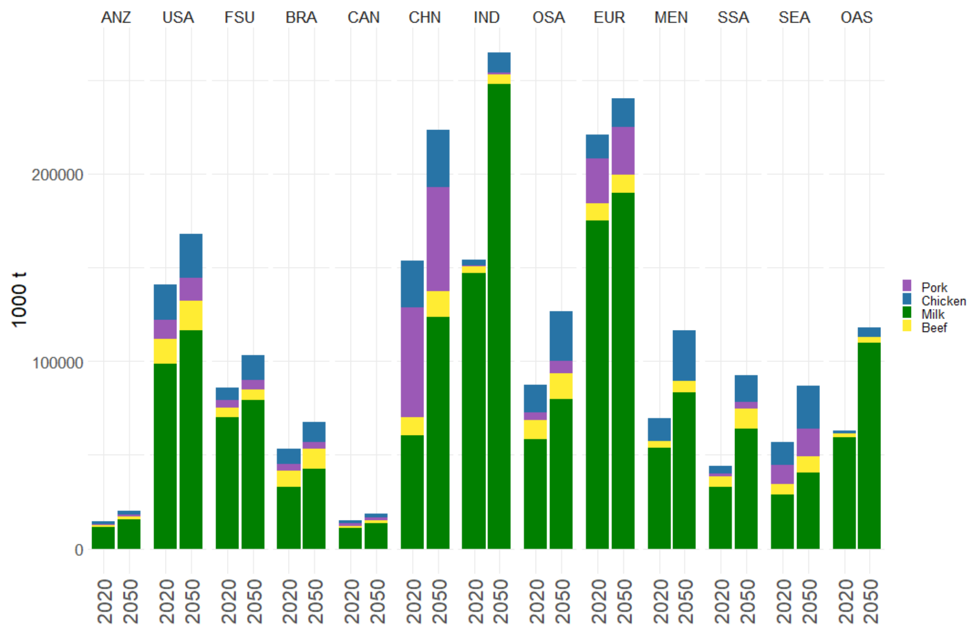

Figure S3 Domestic use of the main ASF in 2020 and 2050 REF

Regional abbreviations: Brazil (BRA), China (CHN), Former Soviet Union (FSU), India (IND), Middle East and North Africa (MEN), Other Asia (OAS), Other South America (OSA), Southeast Asia (SEA), Sub-Saharan Africa (SSA), Oceania (ANZ), Canada (CAN), Europe (EUR), United States (USA).

Table S1 Global shares of individual recipes in total product consumption in 2050 across substitution scenarios

	10% All	25% All	50% All	90% All
Pork R1	1%	2%	4%	7%
Pork R2	9%	23%	46%	83%
Chicken R1	0%	0%	1%	4%
Chicken R2	10%	25%	49%	86%
Milk R1	8%	21%	42%	75%
Milk R2	2%	4%	8%	15%

Beef recipes 1-4 (R1-R4) are not selected and as a result only Beef subst. R5 is the only substitute. All the other dimensions of the scenarios were kept constant (global scope, free sourcing, inefficient processing)

Supplementary notes 2 Differences in the environmental footprint between the ASF products

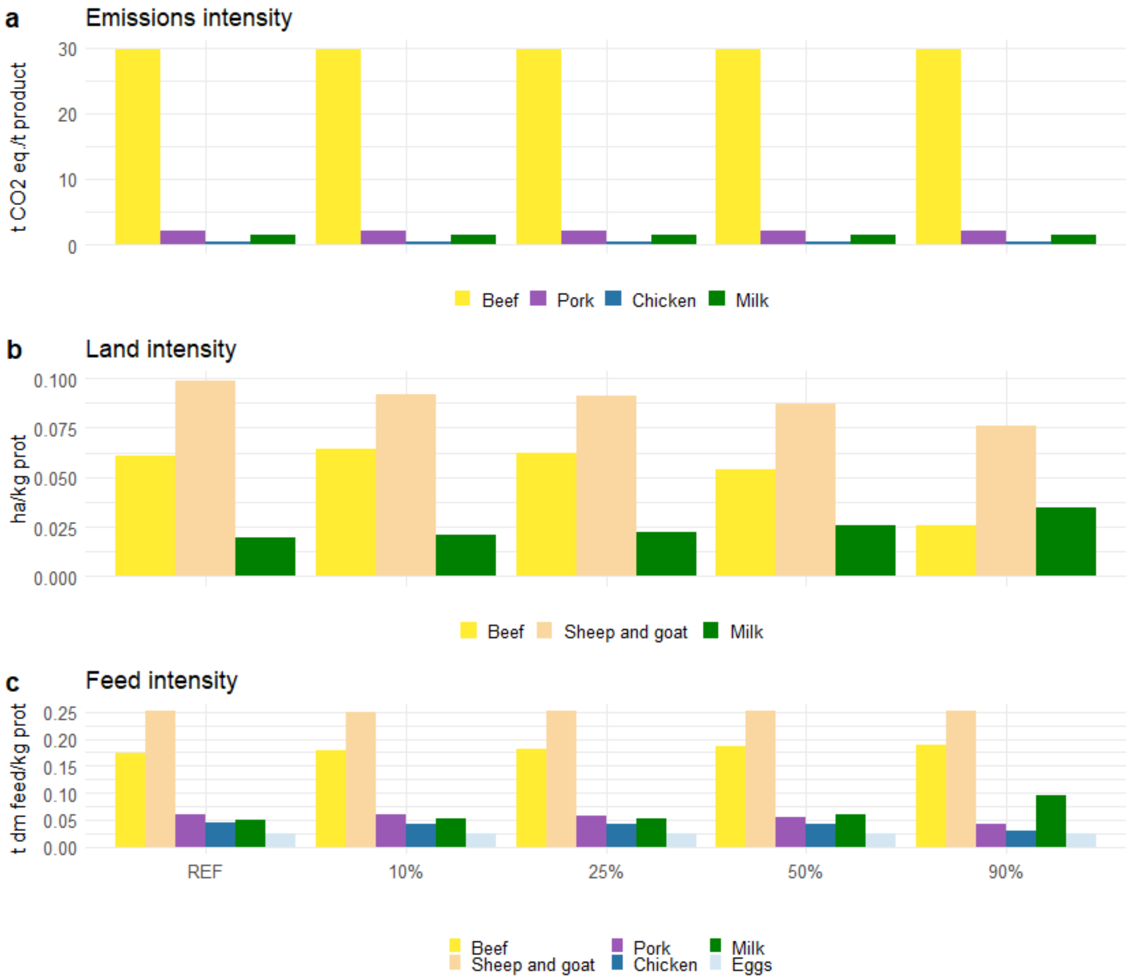

Figure S4 Global average ASF emissions, land, and feed intensity across substitution rates in 2050

In (a), (b) and (c) scenarios assume all product global substitution. Key: prot-protein. Key: REF-reference scenario

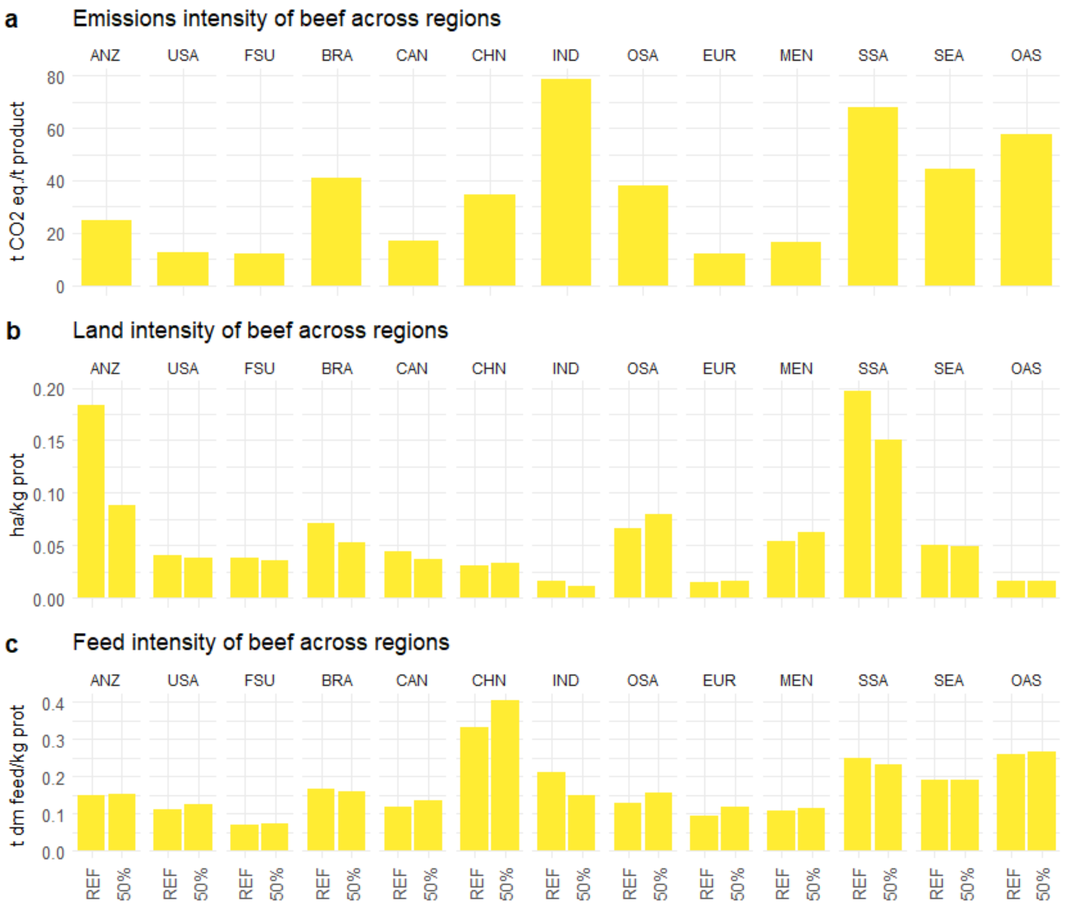

Figure S5 Beef regional average ASF emissions, land, and feed intensity in REF and 50% substitution – all product global substitution scenario in 2050

Regional abbreviations: Brazil (BRA), China (CHN), Former Soviet Union (FSU), India (IND), Middle East and North Africa (MEN), Other Asia (OAS), Other South America (OSA), Southeast Asia (SEA), Sub-Saharan Africa (SSA), Oceania (ANZ), Canada (CAN), Europe (EUR), United States (USA). Key: prot-protein; REF-reference scenario

Supplementary notes 3 Biodiversity Intactness Index – global trends

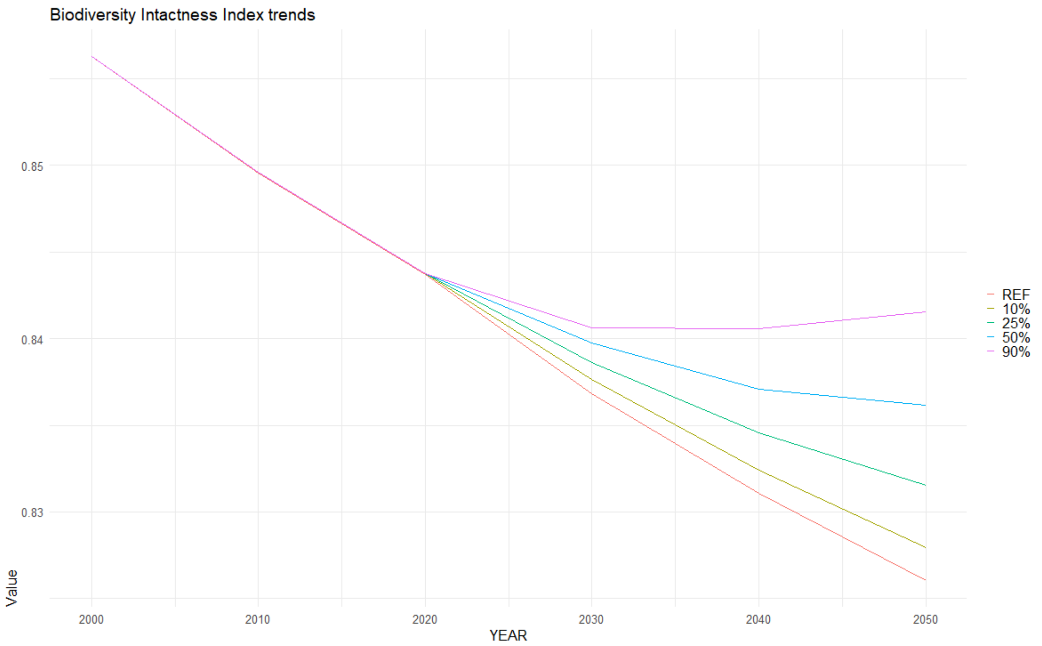

Figure S6 Global Biodiversity Intactness Index time series across scenarios (REF, 10%, 25%, 50% and 90% substitution globally)

Scenarios assume global substitution scope, all products, free trade sourcing, inefficient processing with land restoration on the abandoned agricultural land. Land restoration was modeled as afforestation only within former forest ecosystems and with locally naturally occurring tree species. Key: REF-reference scenario

Supplementary notes 4 Inefficient vs. efficient processing - differences across outcomes

Abbreviations in the tables stand for INE- inefficient processing, EFF- efficient processing

Table S2 Food systems impacts globally in 2050

	Crop use novel alternatives			Crop use total			Crop price change			UNDN		
	Mt		% diff	Mt		% diff	%		pp diff	Mln people		% diff
**Scenario**	**EFF**	**INE**		**EFF**	**INE**		**EFF**	**INE**		**EFF**	**INE**	
10%	21	53	-59%	4755	4784	-1%	-4.4	-4.2	-0.2	347	348	-0.3%
25%	52	132	-61%	4477	4551	-2%	-5.1	-4.9	-0.3	336	339	-0.9%
50%	103	264	-61%	4010	4166	-4%	-6.2	-5.7	-0.6	324	326	-0.6%
90%	184	477	-61%	3361	3647	-8%	-7.5	-6.3	-1.3	310	313	-1.1%

Key: pp-percentage point

Table S3 Land use impacts globally in 2050

	Cropland			Forest			Grassland			Natural land			Restored land		
	Mha		% diff	Mha		% diff	Mha		% diff	Mha		% diff	Mha		% diff
**Scenario**	**EFF**	**INE**		**EFF**	**INE**		**EFF**	**INE**		**EFF**	**INE**		**EFF**	**INE**	
10%	1779	1783	-0.2%	3364	3363	0.0%	3376	3376	0.0%	1803	1802	0.0%	2382	2380	0.1%
25%	1747	1760	-0.7%	3398	3397	0.0%	3153	3149	0.1%	1850	1849	0.0%	2556	2549	0.3%
50%	1677	1706	-1.7%	3424	3423	0.0%	2718	2714	0.1%	1902	1898	0.2%	2983	2963	0.7%
90%	1557	1616	-3.7%	3438	3438	0.0%	1977	1971	0.3%	1997	1987	0.5%	3734	3692	1.1%

Table S4 GHG emissions impacts globally in 2050

Scenario	Agricultural NonCO2 Mt CO2 eq/year EFF	Agricultural NonCO2 Mt CO2 eq/year INE	Agricultural NonCO2 % diff	Agricultural CO2 Mt CO2 eq/year EFF	Agricultural CO2 Mt CO2 eq/year INE	Agricultural CO2 % diff	Forestry CO2 Mt CO2 eq/year EFF	Forestry CO2 Mt CO2 eq/year INE	Forestry CO2 % diff
10%	6362	6364	0.0%	1136	1141	-0.4%	-761	-749	1.6%
25%	5676	5685	-0.1%	808	809	-0.1%	-1710	-1672	2.3%
50%	4462	4482	-0.4%	498	504	-1.2%	-3926	-3814	2.9%
90%	2452	2483	-1.3%	425	448	-5.2%	-7597	-7359	3.2%

Table S5 Biodiversity Intactness Index and agricultural inputs use impacts in 2050

Scenario	BII % change to 2020 EFF	BII % change to 2020 INE	BII pp diff	Nitrogen use Mt EFF	Nitrogen use Mt INE	Nitrogen use % diff	Water use km3 EFF	Water use km3 INE	Water use % diff
10%	-1.9	-1.9	0.0	186	186	-0.4%	3108	3130	-0.7%
25%	-1.4	-1.4	0.0	173	175	-1.0%	2957	3012	-1.8%
50%	-0.8	-0.9	0.1	150	154	-2.4%	2638	2752	-4.2%
90%	-0.2	-0.3	0.1	120	126	-5.2%	2307	2416	-4.5%

Note: Free trade, all product replacement, global implementation assumed. Key: pp-percentage point

Table S6 All vs. individual product scenarios of 50% substitution globally – global impacts, diff to REF in 2050

	Agricultural land	Forest and natural land	Restored land	GHG emissions	BII
	Mha	Mha	Mha	Mt CO2 eq/year	%
**ALL**	-822	200	621	-6279	1.2%
**Beef R1**	-356	131	225	-2920	0.7%
**Beef R2**	-341	123	218	-2847	0.7%
**Beef R3**	-352	130	222	-2880	0.7%
**Beef R4**	-352	129	222	-2897	0.7%
**Beef R5**	-354	130	224	-2915	0.7%
**Chicken R1**	-43	14	29	-329	0.1%
**Chicken R2**	-47	10	31	-324	0.1%
**Pork R1**	-42	16	32	-388	0.0%
**Pork R2**	-43	10	33	-387	0.0%
**Milk R1**	-417	74	342	-2014	0.2%
**Milk R2**	-402	69	334	-1942	0.2%

Note: All the other dimensions of the scenarios were kept constant (global scope, free sourcing, inefficient processing. GHG emissions contain agriculture and land use emissions, including sinks from land restoration. Key: BII – Biodiversity Intactness Index

Supplementary notes 5 Regional crop and ASF availability

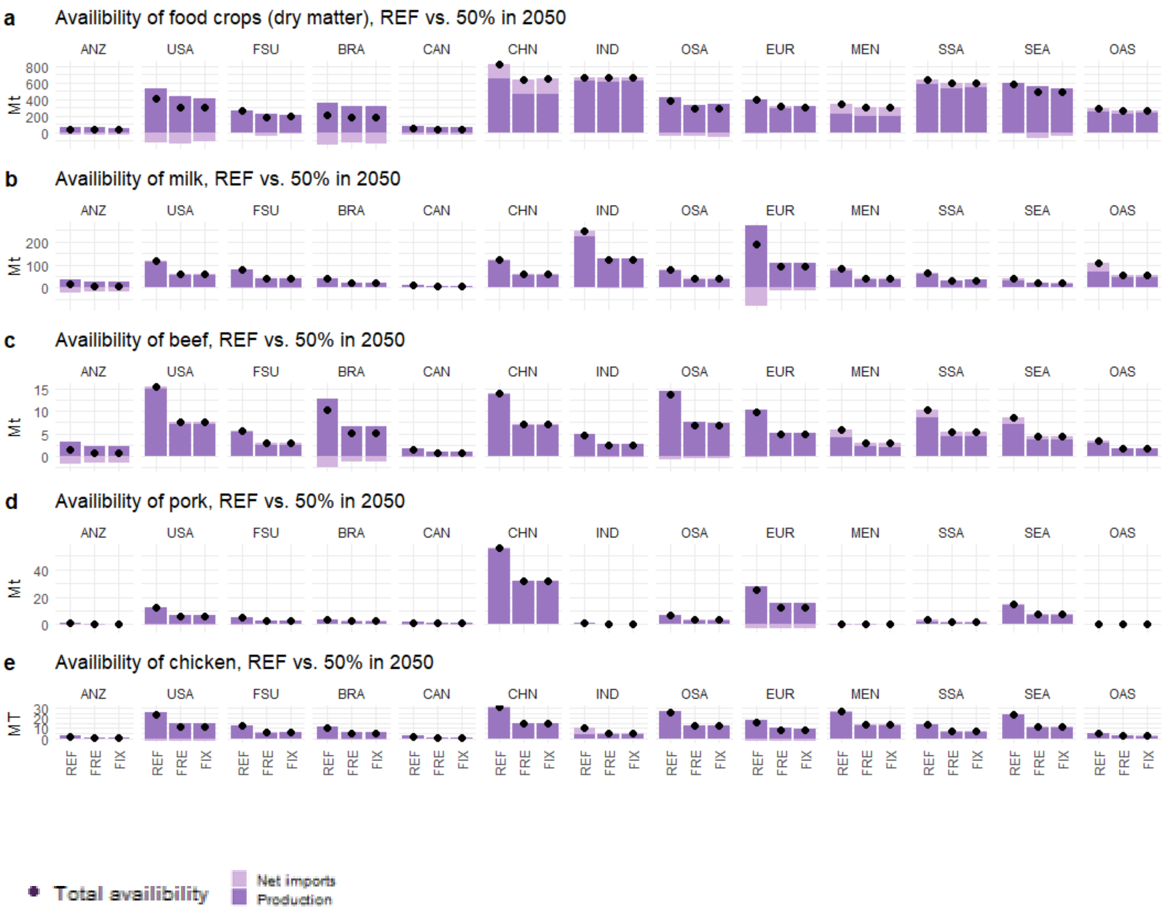

Figure S7 Regional production and trade impacts in agricultural sector - REF and 50% substitution rate scenarios with local (FIX) and free (FRE) sourcing (in 2050)

In (a)-(e) scenarios assume global substitution scope, all products, inefficient processing. Regional abbreviations: Brazil (BRA), China (CHN), Former Soviet Union (FSU), India (IND), Middle East and North Africa (MEN), Other Asia (OAS), Other South America (OSA), Southeast Asia (SEA), Sub-Saharan Africa (SSA), Oceania (ANZ), Canada (CAN), Europe (EUR), United States (USA). In figure (b), Land use CO_2 emissions refer to land use change related directly to agricultural use and do not take into account land restoration impacts, which are represented by Restored land CO_2 category. Key: REF – reference scenario

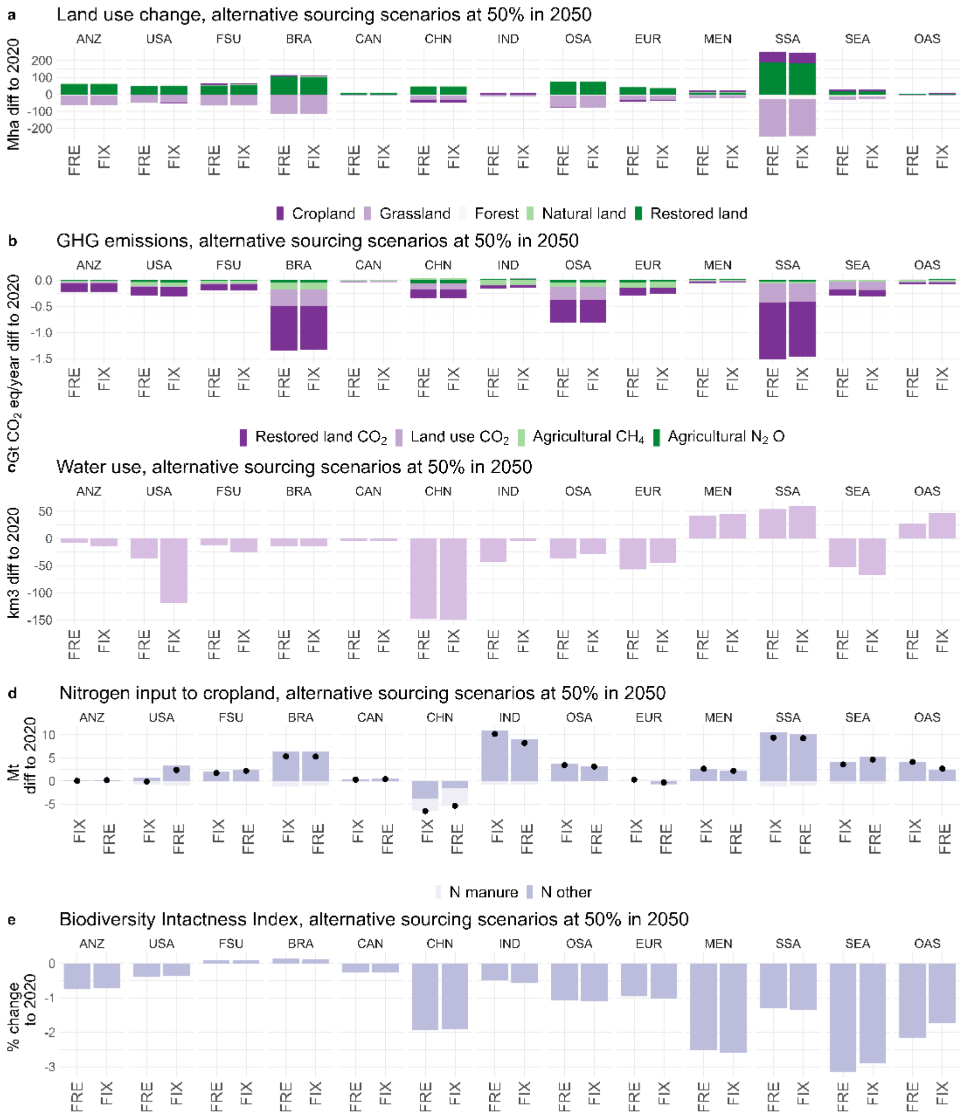

Figure S8 Regional environmental impacts in 2050 across scenarios - 50% with global (FRE) or local (FIX) sourcing

In (a)-(e) scenarios assume global substitution scope, all products, inefficient processing. Regional abbreviations: Brazil (BRA), China (CHN), Former Soviet Union (FSU), India (IND), Middle East and North Africa (MEN), Other Asia (OAS), Other South America (OSA), Southeast Asia (SEA), Sub-Saharan Africa (SSA), Oceania (ANZ), Canada (CAN), Europe (EUR), United States (USA). In figure (b), Land use CO2 emissions refer to land use change related directly to agricultural use and do not take into account land restoration impacts, which are represented by Restored land CO2 category.

Supplementary notes 6 Regional vs. global substitution scenarios – regional distribution of impacts

Table S7 Total crop use, regional impacts in regional vs. global substitution 50% INE scenario, % change to REF 2050

Interv. /Impact	ANZ	USA	FSU	BRA	CAN	CHN	IND	OSA	EUR	MEN	SSA	SEA	OAS	WLD
ANZ	-11.2%	-0.7%	-0.4%	-0.4%	-0.3%	-0.3%	-3.3%	-0.2%	-0.9%	-0.3%	-2.0%	-1.2%	-0.6%	-20.8%
USA	0.0%	-25.3%	0.0%	0.2%	-0.4%	-0.2%	0.2%	0.3%	0.1%	0.0%	0.0%	0.1%	0.0%	-26.2%
FSU	0.0%	-0.1%	-25.5%	0.0%	0.0%	0.4%	-1.5%	0.0%	-2.0%	-0.2%	-0.5%	-0.4%	0.0%	-30.3%
BRA	-0.3%	-0.2%	-0.4%	-10.2%	0.0%	-0.2%	-0.3%	0.1%	-0.6%	0.0%	-0.1%	-0.2%	0.0%	-12.0%
CAN	-0.1%	-0.6%	0.4%	-0.2%	-17.4%	1.2%	-2.0%	-0.1%	-0.6%	-0.2%	-1.5%	-0.3%	-0.1%	-23.5%
CHN	0.0%	0.0%	0.0%	0.0%	0.0%	-20.4%	-0.1%	0.0%	0.0%	0.0%	0.0%	0.0%	0.0%	-20.7%
IND	-0.1%	-0.1%	-0.1%	-0.1%	-0.1%	-0.1%	2.4%	-0.1%	-0.3%	0.0%	-0.1%	-0.1%	-0.1%	2.5%
OSA	-0.2%	0.1%	-0.3%	-0.1%	-0.1%	-0.2%	-1.0%	-21.7%	-0.9%	-0.3%	-0.6%	-0.3%	-0.3%	-24.5%
EUR	0.0%	0.0%	0.0%	0.0%	0.0%	0.0%	-3.4%	-0.2%	-14.6%	-0.3%	-1.1%	-0.4%	-1.5%	-22.5%
MEN	0.0%	0.0%	0.1%	-0.1%	0.0%	0.1%	-0.4%	-0.1%	0.0%	-10.6%	0.0%	0.0%	0.0%	-11.1%
SSA	-0.1%	-0.1%	0.0%	0.0%	-0.1%	-0.1%	-0.3%	0.0%	-0.7%	0.0%	-3.3%	-0.1%	0.0%	-3.9%
SEA	0.0%	0.2%	-0.1%	0.0%	0.0%	-0.3%	-1.0%	-0.1%	-0.3%	-0.1%	-0.3%	-12.8%	-0.1%	-15.2%
OAS	0.0%	0.1%	0.1%	0.0%	0.0%	0.0%	-0.9%	0.2%	0.0%	-0.1%	-0.1%	0.1%	-4.9%	-6.2%

Intervention region (Interv.) – region where substitution scenario is introduced (columns) ; Impact region – region where the impacts are assessed (rows)

Table S8 Undernourishment, regional impacts in regional vs. global substitution 50% INE scenario, M ppl diff to global substitution scenario 2050

Interv. /Impact	ANZ	USA	FSU	BRA	CAN	CHN	IND	OSA	EUR	MEN	SSA	SEA	OAS
FSU	0.7	0.7	0.2	0.7	0.7	0.7	0.6	0.7	0.6	0.7	0.6	0.7	0.7
BRA	1.0	0.7	1.0	0.1	0.9	1.0	0.9	0.7	0.7	0.7	0.8	0.7	0.9
CHN	6.0	6.0	5.9	6.2	6.2	0.3	6.0	6.0	6.0	6.1	6.0	6.0	6.0
IND	-0.6	-0.8	-0.6	-0.6	-0.6	-0.6	0.8	-0.7	-0.8	-0.7	-0.6	-0.8	-0.8
OSA	0.6	0.5	0.6	0.5	0.6	0.6	0.6	0.3	0.5	0.5	0.4	0.5	0.6
MEN	0.9	0.8	0.7	1.0	0.8	0.7	0.8	0.9	0.7	0.4	0.7	0.8	0.9
SSA	17.0	16.6	15.4	17.1	17.1	16.6	16.5	16.5	16.1	16.4	5.9	16.6	17.0
SEA	4.8	4.6	4.8	4.9	4.9	4.7	4.8	4.8	4.6	4.8	4.5	0.6	4.9
OAS	0.6	0.5	0.6	0.6	0.6	0.6	0.6	0.5	0.4	0.7	0.6	0.5	0.1

Intervention region (Interv.) – region where substitution scenario is introduced (columns) ; Impact region – region where the impacts are assessed (rows)

Table S9 Cropland, regional impacts in regional vs. global substitution 50% INE scenario, % diff to REF in 2050

Interv. /Impact	ANZ	USA	FSU	BRA	CAN	CHN	IND	OSA	EUR	MEN	SSA	SEA	OAS	WLD
ANZ	0%	0%	-1%	0%	0%	-1%	0%	0%	0%	0%	0%	0%	0%	-3%
USA	0%	0%	0%	0%	0%	0%	0%	0%	0%	0%	0%	0%	0%	-1%
FSU	0%	0%	-1%	0%	0%	0%	0%	0%	0%	0%	0%	0%	0%	-3%
BRA	0%	-1%	0%	1%	0%	3%	0%	-1%	0%	0%	0%	-1%	0%	-5%
CAN	0%	0%	0%	0%	1%	-1%	0%	0%	0%	0%	0%	0%	0%	-4%
CHN	0%	0%	0%	0%	0%	-12%	0%	0%	0%	0%	0%	0%	0%	-13%
IND	0%	0%	0%	0%	0%	0%	0%	0%	0%	0%	0%	0%	0%	0%
OSA	0%	-2%	0%	-1%	0%	0%	-1%	-5%	-1%	0%	-1%	-1%	0%	-12%
EUR	0%	0%	-2%	0%	0%	-1%	-1%	0%	-2%	-1%	-1%	0%	0%	-13%
MEN	0%	0%	0%	0%	0%	0%	0%	0%	0%	0%	0%	0%	0%	-2%
SSA	0%	0%	0%	0%	0%	0%	0%	0%	0%	0%	2%	0%	0%	0%
SEA	0%	0%	0%	0%	0%	0%	0%	0%	0%	0%	-1%	-5%	0%	-5%
OAS	0%	0%	0%	0%	0%	0%	0%	0%	0%	0%	0%	0%	-5%	-6%

Intervention region (Interv.) – region where substitution scenario is introduced (columns) ; Impact region – region where the impacts are assessed (rows)

Table S10 Grassland, regional impacts in regional vs. global substitution 50% INE scenario, % diff to REF in 2050

Interv. /Impact	ANZ	USA	FSU	BRA	CAN	CHN	IND	OSA	EUR	MEN	SSA	SEA	OAS	WLD
**ANZ**	-9%	-2%	-1%	-2%	-1%	-2%	-2%	-2%	-2%	-2%	-2%	-2%	-2%	-14%
**USA**	0%	-20%	0%	0%	0%	0%	0%	0%	0%	0%	0%	0%	0%	-22%
**FSU**	0%	0%	-15%	0%	0%	0%	0%	0%	0%	0%	0%	0%	0%	-15%
**BRA**	0%	0%	0%	-49%	0%	0%	0%	0%	0%	0%	-1%	0%	0%	-54%
**CAN**	0%	-2%	0%	0%	-39%	0%	0%	0%	0%	-1%	0%	0%	0%	-42%
**CHN**	0%	0%	0%	0%	0%	-8%	0%	0%	0%	0%	0%	0%	0%	-8%
**IND**	-2%	-2%	-2%	-2%	-2%	-1%	-32%	-2%	-1%	-2%	-2%	-2%	-2%	-31%
**OSA**	0%	0%	0%	0%	0%	0%	0%	-15%	0%	0%	0%	0%	0%	-19%
**EUR**	0%	0%	0%	0%	0%	0%	-1%	-1%	-28%	-1%	-1%	-1%	-1%	-34%
**MEN**	-2%	-2%	-2%	-2%	-2%	-2%	-2%	-2%	-2%	-6%	-2%	-2%	-2%	-6%
**SSA**	-1%	-1%	-1%	-1%	-1%	-1%	-1%	-1%	-1%	-1%	-30%	-1%	-1%	-32%
**SEA**	0%	0%	0%	0%	0%	0%	0%	0%	0%	0%	0%	-15%	0%	-16%
**OAS**	-2%	-2%	-2%	-2%	-2%	-2%	-2%	-2%	-2%	-2%	-2%	-2%	-14%	-14%

Intervention region (Interv.) – region where substitution scenario is introduced (columns) ; Impact region – region where the impacts are assessed (rows)

Table S11 Natural land, regional impacts in regional vs. global substitution 50% INE scenario, % diff to REF in 2050

Interv. /Impact	ANZ	USA	FSU	BRA	CAN	CHN	IND	OSA	EUR	MEN	SSA	SEA	OAS	WLD
**ANZ**	0%	0%	0%	0%	0%	0%	0%	0%	0%	0%	0%	0%	0%	3%
**USA**	0%	2%	0%	0%	0%	0%	0%	0%	0%	0%	0%	0%	0%	2%
**FSU**	0%	0%	1%	0%	0%	0%	0%	0%	0%	0%	0%	0%	0%	1%
**BRA**	0%	0%	0%	4%	0%	0%	0%	0%	0%	0%	0%	0%	0%	5%
**CAN**	0%	0%	0%	0%	0%	0%	0%	0%	0%	0%	0%	0%	0%	0%
**CHN**	-1%	-1%	-1%	-1%	-1%	8%	-1%	-1%	-1%	-1%	-1%	0%	-1%	8%
**IND**	1%	1%	1%	1%	1%	1%	12%	1%	1%	1%	1%	1%	1%	12%
**OSA**	0%	0%	0%	0%	0%	0%	0%	3%	0%	0%	0%	0%	0%	3%
**EUR**	0%	0%	1%	0%	0%	0%	1%	0%	4%	1%	1%	0%	1%	5%
**MEN**	18%	18%	19%	18%	18%	19%	19%	19%	19%	47%	19%	19%	18%	51%
**SSA**	0%	0%	0%	0%	0%	0%	0%	0%	0%	0%	8%	0%	0%	9%
**SEA**	0%	0%	0%	0%	0%	0%	0%	0%	0%	0%	0%	7%	0%	8%
**OAS**	1%	1%	1%	1%	1%	1%	1%	1%	1%	1%	1%	1%	4%	5%

Intervention region (Interv.) – region where substitution scenario is introduced (columns) ; Impact region – region where the impacts are assessed (rows)

Note: Natural land= forest+ other natural land

Table S12 Land use CO^2 emissions, regional impacts in regional vs. global substitution 50% INE scenario, Mt CO2eq/year, diff to REF

Interv. /Impact	ANZ	USA	FSU	BRA	CAN	CHN	IND	OSA	EUR	MEN	SSA	SEA	OAS	WLD
**ANZ**	10	14	16	15	15	16	15	15	16	15	15	11	15	4
**USA**	0	-31	0	0	0	0	0	0	0	0	0	0	0	-32
**FSU**	1	1	-1	1	0	0	0	1	0	1	0	1	0	-3
**BRA**	1	2	3	-66	1	16	0	0	-1	0	-27	1	1	-83
**CAN**	0	0	0	0	-4	0	0	0	0	0	0	0	0	-3
**CHN**	1	2	1	2	2	-61	1	1	1	1	1	0	1	-61
**IND**	-1	-2	0	0	0	0	-23	-1	0	-1	0	-4	-3	-23
**OSA**	-1	-12	-1	-14	-2	1	-6	-69	-9	-17	-14	-14	-8	-100
**EUR**	-3	-5	-7	-3	-3	-6	-5	-5	-16	-5	-7	-4	-7	-29
**MEN**	-1	-1	-3	-1	-1	-1	-1	0	-3	-10	0	0	-1	-13
**SSA**	10	5	6	10	9	8	7	6	8	7	-287	10	9	-329
**SEA**	-2	-2	-1	-3	-3	-5	-1	-1	-2	-2	-4	-121	-2	-132
**OAS**	-1	-1	-1	-1	-1	-1	-1	0	-1	-1	-2	-1	-8	-8

Intervention region (Interv.) – region where substitution scenario is introduced (columns) ; Impact region – region where the impacts are assessed (rows)

Note: Natural land= forest+ other natural land

Table S13 Agricultural non-CO^2 emissions, regional impacts in regional vs. global substitution 50% INE scenario, Mt CO2eq/year, diff to REF

Interv. /Impact	ANZ	USA	FSU	BRA	CAN	CHN	IND	OSA	EUR	MEN	SSA	SEA	OAS	WLD
**ANZ**	-22	3	2	1	3	2	2	3	1	2	1	-3	-2	-45
**USA**	1	-168	0	2	-1	0	3	1	2	0	0	-1	0	-178
**FSU**	0	-1	-104	0	0	0	-2	0	-4	-1	-1	-2	0	-114
**BRA**	-1	-1	-1	-186	-1	0	-1	-10	-1	-7	-13	-2	-1	-227
**CAN**	0	-1	0	0	-16	0	0	0	0	0	-1	0	0	-20
**CHN**	5	5	5	5	6	-456	5	4	4	5	4	2	6	-463
**IND**	-4	-6	-5	-4	-4	-5	-201	-6	-7	-6	-6	-8	-7	-216
**OSA**	2	2	2	0	2	2	1	-181	0	-2	-8	0	0	-246
**EUR**	-1	-2	-7	-2	-1	-3	-20	-5	-169	-6	-8	-5	-14	-242
**MEN**	0	0	0	0	1	1	0	0	0	-45	0	0	0	-53
**SSA**	-3	-3	-3	-3	-3	-3	-3	-5	-4	-4	-154	-4	-3	-175
**SEA**	0	1	-1	0	0	-2	-1	-1	-2	-4	-4	-185	-1	-201
**OAS**	-2	-2	-2	-2	-2	-2	-3	-3	-3	-2	-3	-2	-56	-61

Intervention region (Interv.) – region where substitution scenario is introduced (columns) ; Impact region – region where the impacts are assessed (rows)

Note: Natural land= forest+ other natural land

Table S14 CO^2 sinks from restored land, regional impacts in regional vs. global substitution 50% INE scenario, Mt CO2eq/year, diff to REF

Interv. /Impact	ANZ	USA	FSU	BRA	CAN	CHN	IND	OSA	EUR	MEN	SSA	SEA	OAS	WLD
**ANZ**	-114	-22	-22	-22	-22	-23	-23	-23	-23	-23	-24	-26	-31	-148
**USA**	-1	-145	-1	-1	-1	-1	-1	-1	-1	-1	-1	-1	0	-166
**FSU**	0	0	-105	0	0	0	0	0	0	0	0	0	0	-114
**BRA**	0	-4	1	-736	-1	0	-1	-6	-1	-3	-5	-2	1	-833
**CAN**	0	0	0	0	-7	-1	0	0	0	0	0	0	0	-12
**CHN**	1	0	0	1	1	-132	1	0	0	0	0	-2	1	-142
**IND**	-2	-2	-2	-2	-2	-2	-50	-2	-2	-2	-2	-2	-2	-55
**OSA**	-5	-11	-5	-6	-5	-4	-6	-253	-6	-6	-8	-6	-4	-393
**EUR**	0	-1	-6	0	0	-2	-2	-1	-72	-3	-2	-1	-2	-133
**MEN**	-2	-2	-2	-2	-2	-2	-2	-2	-2	-23	-2	-2	-2	-28
**SSA**	-26	-26	-25	-26	-26	-27	-27	-38	-25	-27	-943	-32	-27	-1063
**SEA**	0	0	0	0	0	0	0	0	0	-1	-2	-92	0	-102
**OAS**	-1	-1	-1	-1	-1	-1	-1	-1	-1	-1	-1	-1	-29	-30

Intervention region (Interv.) – region where substitution scenario is introduced (columns) ; Impact region – region where the impacts are assessed (rows)

Note: Natural land= forest+ other natural land

Table S15 Water use, regional impacts in regional vs. global substitution 50% INE scenario, % diff to REF

Interv. /Impact	ANZ	USA	FSU	BRA	CAN	CHN	IND	OSA	EUR	MEN	SSA	SEA	OAS	WLD
**ANZ**	-6%	-8%	-1%	-2%	1%	-1%	-3%	-4%	-3%	0%	-3%	-6%	-1%	-36%
**USA**	-2%	-6%	-5%	-2%	-2%	2%	-2%	-5%	-2%	-2%	-1%	-4%	0%	-9%
**FSU**	4%	7%	-19%	8%	6%	-2%	-4%	3%	-10%	-5%	-5%	4%	8%	-33%
**BRA**	4%	-3%	3%	-1%	6%	-5%	4%	-8%	-2%	-2%	-1%	-8%	1%	14%
**CAN**	-1%	-80%	-87%	-1%	-4%	-85%	0%	-56%	-1%	-13%	-27%	0%	-1%	-90%
**CHN**	1%	1%	1%	1%	1%	-33%	0%	1%	1%	1%	1%	1%	1%	-33%
**IND**	-1%	-1%	-1%	-1%	-1%	-1%	1%	-1%	-1%	-1%	-1%	-2%	-1%	-3%
**OSA**	-1%	-7%	-2%	-1%	-1%	-1%	-1%	-20%	-6%	-2%	-3%	-3%	-1%	-33%
**EUR**	3%	1%	-42%	4%	3%	0%	2%	0%	13%	4%	1%	5%	4%	-41%
**MEN**	1%	1%	0%	1%	1%	1%	1%	1%	0%	-9%	1%	1%	1%	-14%
**SSA**	1%	0%	0%	1%	1%	0%	0%	0%	-1%	-2%	-2%	0%	1%	-12%
**SEA**	0%	0%	0%	0%	0%	0%	-1%	0%	0%	-1%	-1%	-10%	0%	-13%
**OAS**	1%	1%	0%	0%	0%	0%	0%	1%	0%	0%	0%	0%	-3%	-3%

Intervention region (Interv.) – region where substitution scenario is introduced (columns) ; Impact region – region where the impacts are assessed (rows)

Note: Natural land= forest+ other natural land

Table S16 Nitrogen use, regional impacts in regional vs. global substitution 50% INE scenario, % diff to REF

Interv. /Impact	ANZ	USA	FSU	BRA	CAN	CHN	IND	OSA	EUR	MEN	SSA	SEA	OAS	WLD
ANZ	0%	1%	-3%	0%	0%	-3%	-2%	0%	-3%	-1%	-1%	0%	0%	-9%
USA	0%	-14%	-1%	0%	0%	0%	0%	-3%	0%	0%	0%	-1%	0%	-21%
FSU	0%	-1%	-7%	0%	0%	0%	-1%	0%	-2%	-1%	-1%	-1%	0%	-12%
BRA	0%	-2%	0%	-10%	0%	2%	-1%	-1%	-1%	-3%	0%	-3%	0%	-22%
CAN	0%	-1%	0%	0%	0%	-3%	0%	0%	0%	0%	-1%	0%	0%	-12%
CHN	0%	0%	0%	0%	0%	-22%	0%	0%	0%	0%	0%	0%	0%	-23%
IND	0%	0%	0%	0%	0%	0%	-1%	0%	-1%	0%	0%	-1%	0%	-4%
OSA	0%	-1%	0%	-1%	0%	-1%	-1%	-30%	-2%	-1%	-10%	-1%	0%	-49%
EUR	0%	-1%	-5%	0%	0%	-2%	-3%	-1%	-9%	-3%	-2%	-1%	-1%	-30%
MEN	0%	0%	0%	0%	1%	1%	0%	0%	-1%	-5%	0%	0%	0%	-11%
SSA	0%	0%	-1%	0%	0%	0%	0%	-2%	-1%	0%	-1%	-1%	0%	-8%
SEA	0%	0%	0%	0%	0%	0%	-1%	0%	0%	0%	-1%	-17%	0%	-22%
OAS	0%	0%	0%	0%	0%	0%	-1%	0%	0%	0%	0%	0%	-10%	-12%

Intervention region (Interv.) – region where substitution scenario is introduced (columns) ; Impact region – region where the impacts are assessed (rows)

Note: Natural land= forest+ other natural land

Table S17 Biodiversity Intactness Index, regional impacts in regional vs. global substitution 50% INE scenario, pp. diff to REF

Interv. /Impact	ANZ	USA	FSU	BRA	CAN	CHN	IND	OSA	EUR	MEN	SSA	SEA	OAS	WLD
ANZ	-0.3	-0.1	-0.1	-0.1	-0.1	-0.1	-0.1	-0.1	-0.1	-0.1	-0.1	-0.1	-0.1	-0.2
USA	0.0	0.4	0.0	0.0	0.0	0.0	0.0	0.0	0.0	0.0	0.0	0.0	0.0	0.4
FSU	0.0	0.0	0.5	0.0	0.0	0.0	0.0	0.0	0.0	0.0	0.0	0.0	0.0	0.5
BRA	0.0	0.1	0.0	1.1	0.0	-0.2	0.0	0.0	0.0	0.0	0.1	0.1	0.0	1.3
CAN	0.0	0.0	0.0	0.0	0.1	0.0	0.0	0.0	0.0	0.0	0.0	0.0	0.0	0.1
CHN	-0.1	-0.1	0.0	-0.1	-0.1	1.9	0.0	0.0	0.0	0.0	0.0	0.0	-0.1	1.9
IND	0.0	0.0	0.0	0.0	0.0	0.0	1.2	0.0	0.0	0.0	0.0	0.0	0.0	1.3
OSA	0.0	0.1	0.0	0.0	0.0	0.0	0.0	0.7	0.0	0.1	0.1	0.1	0.0	1.1
EUR	0.0	0.1	0.1	0.0	0.0	0.1	0.1	0.1	1.4	0.1	0.1	0.1	0.1	2.0
MEN	0.0	0.0	0.0	0.0	0.0	0.0	0.0	0.0	0.0	0.9	0.0	0.0	0.0	1.0
SSA	0.1	0.1	0.1	0.1	0.1	0.1	0.1	0.1	0.1	0.1	1.7	0.1	0.1	2.0
SEA	0.0	0.0	0.0	0.0	0.0	0.0	-0.1	0.0	0.0	0.0	0.1	2.0	0.0	2.1
OAS	0.0	0.0	0.1	0.0	0.0	0.0	0.0	0.0	-0.4	0.0	0.0	0.1	1.0	0.8

Intervention region (Interv.) – region where substitution scenario is introduced (columns) ; Impact region – region where the impacts are assessed (rows)

Note: Natural land= forest+ other natural land

Supplementary notes 7 Regional country mapping

CAN: Canada
USA: USA, Puerto Rico

BRA: Brazil

OSA: Argentina, Bahamas, Belize, Bolivia, Chile, Colombia, Costa Rica, Cuba, Dominican Rp, Ecuador, El Salvador, Falkland Is, Fr Guiana, Guadeloupe, Guatemala, Guyana, Haiti, Honduras, Jamaica, Mexico, Nicaragua, Panama, Paraguay, Peru, Suriname, Trinidad Tob, Uruguay, Venezuela

FSU: Armenia, Azerbaijan, Georgia, Kazakhstan, Tajikistan, Kyrgyzstan, Moldova Rep, Russian Fed, Turkmenistan, Ukraine, Uzbekistan, Belarus

EUR: Albania, Austria, Belgium, Bosnia and Herzegovina, Bulgaria, Croatia, Cyprus, Czech Rep, Denmark, Estonia, Finland, France, Germany, Greece, Greenland, Hungary, Iceland, Ireland, Italy,

Latvia, Lithuania, Luxembourg, Macedonia, Malta, Montenegro, Netherlands, Norway, Poland, Portugal, Romania, Serbia, Slovakia, Slovenia, Spain, Sweden, Switzerland, UK

MEN: Algeria, Bahrain, Egypt, Iraq, Israel, Jordan, Kuwait, Lebanon, Libya, Morocco, Oman, Palestine, Qatar, Saudi Arabia, Syria, Tunisia, Turkey, United Arab Emirates, West Sahara, Yemen, Iran

SSA: Angola, Benin, Botswana, Cote d'Ivoire, Burkina Faso, Burundi, Cameroon, Cape Verde, Cent African Republic, Chad, Comoros, Congo Democratic Republic, Congo Republic, Djibouti, Equatorial Guinea, Eritrea, Ethiopia, Gabon, Gambia, Ghana, Guinea, Guinea Bissau, Kenya, Lesotho, Liberia, Madagascar, Malawi, Mali, Mauritania, Mauritius, Mozambique, Namibia, Niger, Nigeria, Reunion, Rwanda, Senegal, Sierra Leone, Somalia, South Africa, Sudan, Swaziland, Tanzania, Togo, Uganda, Zambia, Zimbabwe

CHN: China

IND: India

SEA: Brunei Darussalam, Cambodia, Indonesia, Japan, Korea DPR, Laos, Malaysia, Mongolia, Myanmar, Philippines, Singapore, Thailand, Timor-Leste, Vietnam

OAS: Afghanistan, Bangladesh, Bhutan, Fiji Islands, French Polynesia, Nepal, New Caledonia, Pakistan, Papua New Guinea, Samoa, Solomon Islands, Sri Lanka, Vanuatu

ANZ: Australia, New Zealand

Supplementary notes 8 Receipts composition details and processing conversions of processed products and by-products

While the choice of the oils for the standard trade scenario was guided by the most widely used oil in the region, oils used in the local sourcing (FIX) scenario were selected from the most domestically supplied ones (table s18). The method used to derive top domestic oil was based on baseline 2020 GLOBIOM projections of crop production per region and multiply that with the respective oil yields and the percentage of the given crop that is currently globally processed into oils/meals. We also confronted the 2020 GLOBIOM projections with statistics from FAO Food Balance Sheets and USDA Production, Supply, and Distribution for the latest available year (2018 in FAOSTAT, 2018/2019 in USDA). This check included information on the trade of the actual oils as well as the underlying oilseeds. This helped to identify the most widely used oil, regardless of whether it's imported or domestic.

Table S18 Preferred vegetable oils in GLOBIOM Regions

	**Most widely-used oil**	**Top domestic oil**
Argentina	Soybean	Soybean
Australia	Rapeseed	Rapeseed
Brazil	Soybean	Soybean
Canada	Rapeseed	Rapeseed
China	Soybean	Rapeseed
Congo Basin	Palm	Palm
Eastern Africa	Palm	Sunflower
EU Baltic	Rapeseed	Rapeseed

EU Central East	Rapeseed	Rapeseed
EU Mid-West	Rapeseed	Rapeseed
EU North	Rapeseed	Rapeseed
EU South	Palm	Sunflower
Former USSR	Sunflower	Sunflower
India	Palm	Rapeseed
Indonesia	Palm	Palm
Japan	Rapeseed	Soybean
Malaysia	Palm	Palm
Mexico	Soybean	Soybean
Middle East	Palm	Soybean
New Zealand	Rapeseed	Rapeseed
Northern Africa	Soybean	Groundnut
Pacific Islands	Palm	Palm
Rest of Central America	Palm	Palm
Rest of Central and Eastern Europe	Sunflower	Sunflower
Rest of Western Europe	Rapeseed	Rapeseed
Rest of South America	Soybean	Soybean
Rest of South Asia	Palm	Cottonseed
Rest of Other Pacific and Asia	Palm	Palm
Rest of Planned Asia	Palm	Soybean
Russia	Sunflower	Sunflower
South Africa	Palm	Sunflower
Southern Africa	Palm	Groundnut
South Korea	Palm	Soybean
Turkey	Sunflower	Sunflower
Ukraine	Sunflower	Sunflower
USA	Soybean	Soybean
Western Africa	Palm	Palm

Conversion of the primary commodities into novel alternatives ingredients was based on various literature sources depending on the commodity.

Cottonseed: Heuzé V., Tran G., Hassoun P., Bastianelli D., Lebas F., 2019. Cottonseed meal. Feedipedia, a programme by INRAE, CIRAD, AFZ and FAO. https://www.feedipedia.org/node/550 Last updated on February 8, 2019, 14:21

Sugarcane: Heuzé V., Tran G., Archimède H., Renaudeau D., Lessire M., Lebas F., 2015. Sugarcane juice. Feedipedia, a programme by INRAE, CIRAD, AFZ and FAO. https://feedipedia.org/node/560 Last updated on October 8, 2015, 18:31

Oil palm: Heuzé V., Tran G., Sauvant D., Lebas F., 2015. Oil palm fronds and oil palm crop residues. Feedipedia, a programme by INRAE, CIRAD, AFZ and FAO. https://www.feedipedia.org/node/6916 Last updated on June 25, 2015, 11:18

Soybean: Berk, Z. Technology of Production of Edible Flours and Protein Products from Soybeans. Technology of production of edible flours and protein products from soybeans vol. 97 http://www.fao.org/docrep/t0532e/t0532e00.htm#con (1992).

Rapeseed: Östbring, K., Nilsson, K., Ahlström, C., Fridolfsson, A. & Rayner, M. Emulsifying and anti-oxidative properties of proteins extracted from industrially cold-pressed rapeseed press-cake. Foods 9, (2020).

Remaining commodities: USDA. FoodData Central. 2022 https://fdc.nal.usda.gov/.

Table S19 Conversion factors from the primary commodity to novel alternatives ingredients used to develop inefficient and efficient processing assumptions

Product	Maximum use	Minimum use
Chickpea protein	100%	22%
Cottonseed Oil	16%	
Dry beans	89%	
Groundnut oil	75%	48%
Palm Oil	20%	
Peanut flour	75%	52%
Potato Protein Isolate	5%	20%
Rapeseed oil	43%	
Rapeseed Protein Isolate	58%	10%
Sorghum flour	100%	
Soy protein isolate	80%	33%
Sugarcane (cane syrup)	65%	29%
Sunflower oil	40%	
Sweet potato, dried	23%	
Wheat flour	74%	
Wheat protein concentrate	74%	11%

Table S20 GLOBIOM beef recipe compositions per unit of substituted carcass weight meat for efficient and inefficient scenarios

RECIPE 1

	Efficient	Inefficient
Soy protein isolate	0.130	0.492
Rapeseed oil	0.130	0.306
Sweet potato, dried[1]	0.170	0.170

RECIPE 2

	Efficient	Inefficient
Rapeseed Protein Isolate	0.130	2.261
Cottonseed Oil	0.130	0.800
Sugarcane (cane syrup)[2]	0.207	0.207

RECIPE 3

	Efficient	Inefficient
Potato Protein Isolate[3]	0.464	10.708
Palm Oil[4]	0.643	0.643
Peanut flour[5]	0.130	0.252

RECIPE 4

	Efficient	Inefficient
Wheat protein concentrate	0.026	0.319
potato protein concentrate 300	0.186	4.283
Soy protein isolate	0.065	0.246

Sunflower oil	0.130	0.325

RECIPE 5

	Efficient	Inefficient
Soy protein isolate	0.130	0.492
Palm oil	0.643	0.643
Cassava (raw)	0.065	0.065

Table S21 GLOBIOM pork recipe compositions per unit of substituted carcass weight meat for efficient and inefficient scenarios

RECIPE 1

	Efficient	Inefficient
Dry beans[1]	0.110	0.110
Soy protein isolate	0.085	0.320
Sorghum flour	0.026	0.026
Groundnut oil*,[5]	0.173	0.357

RECIPE 2

	Efficient	Inefficient
Soy protein isolate	0.130	0.492
Wheat	0.026	0.035
Palm oil[4]	0.643	0.643

Table S22 GLOBIOM chicken recipe compositions per unit of substituted carcass weight meat for efficient and inefficient scenarios

RECIPE 1

	Efficient	Inefficient
Chickpea protein	0.072	0.325
Soy protein isolate	0.072	0.271
Wheat flour	0.055	0.074
Palm oil*,[4]	0.218	0.218

RECIPE 2

	Efficient	Inefficient
Soy protein isolate	0.110	0.417
Sweet potato[1]	0.239	0.239
Groundnut oil[5]	0.059	0.121

Table S23 GLOBIOM milk recipe compositions per unit of substituted carcass weight meat for efficient and inefficient scenarios

RECIPE 1

	Efficient	Inefficient
Soy protein concentrate	0.070	0.117
Sugar cane[6]	0.106	0.106
Palm oil*,[4]	0.148	0.148

RECIPE 2

	Efficient	Inefficient
Wheat protein concentrate	0.030	0.369
Rapeseed meal	0.070	0.108

Rapeseed oil	0.020	0.047

[1] Conversion is simply from fresh to dry matter, which needs to happen in both the efficient and inefficient cases; therefore, the values are the same.

[2] Sugar represents such a large share of the value, even though it's a small share of the mass, we use the same recipe requirement in the efficient case as in the inefficient case.

[3] We account for the 79% moisture content of fresh potato, even in the efficient case.

[4] The oil represents such a large share of the value, even though it's a small share of the mass, we use the same recipe requirement in the efficient case as in the inefficient case.

[5] Sugar represents such a large share of the value, even though it's a small share of the mass, we use the same recipe requirement in the efficient case as in the inefficient case.

[5] The first conversion accounts for the shells, which needs to occur in both the efficient and inefficient case.

* Note, many oils can be used interchangeably as a source of fat in these recipes: rapeseed, cottonseed, peanut, palm, sunflower, soybean

Supplementary notes 9 Additional GLOBIOM model information

GLOBIOM is a partial-equilibrium model that represents main global land use sectors, including agriculture and forestry. The supply side of the model is built from the bottom-up (spatially explicit land cover, land use, management systems and economic cost information) to the top (regional commodity markets). This detailed structure allows for a rich set of environmental and socio-economic parameters, such as prevalence of under-nourishment, deforestation, water withdrawals, nitrogen fertilizer use, GHG emissions, to be taken into account.

The spatial resolution of the supply side relies on the concept of Simulation Units, which are aggregates of 5 to 30 arcmin pixels belonging to the same altitude, slope, and soil class, and follows country borders. For crops, livestock, and forest products, spatially explicit Leontief production functions covering alternative production systems are parameterized using biophysical models like EPIC or G4M (figure s9).

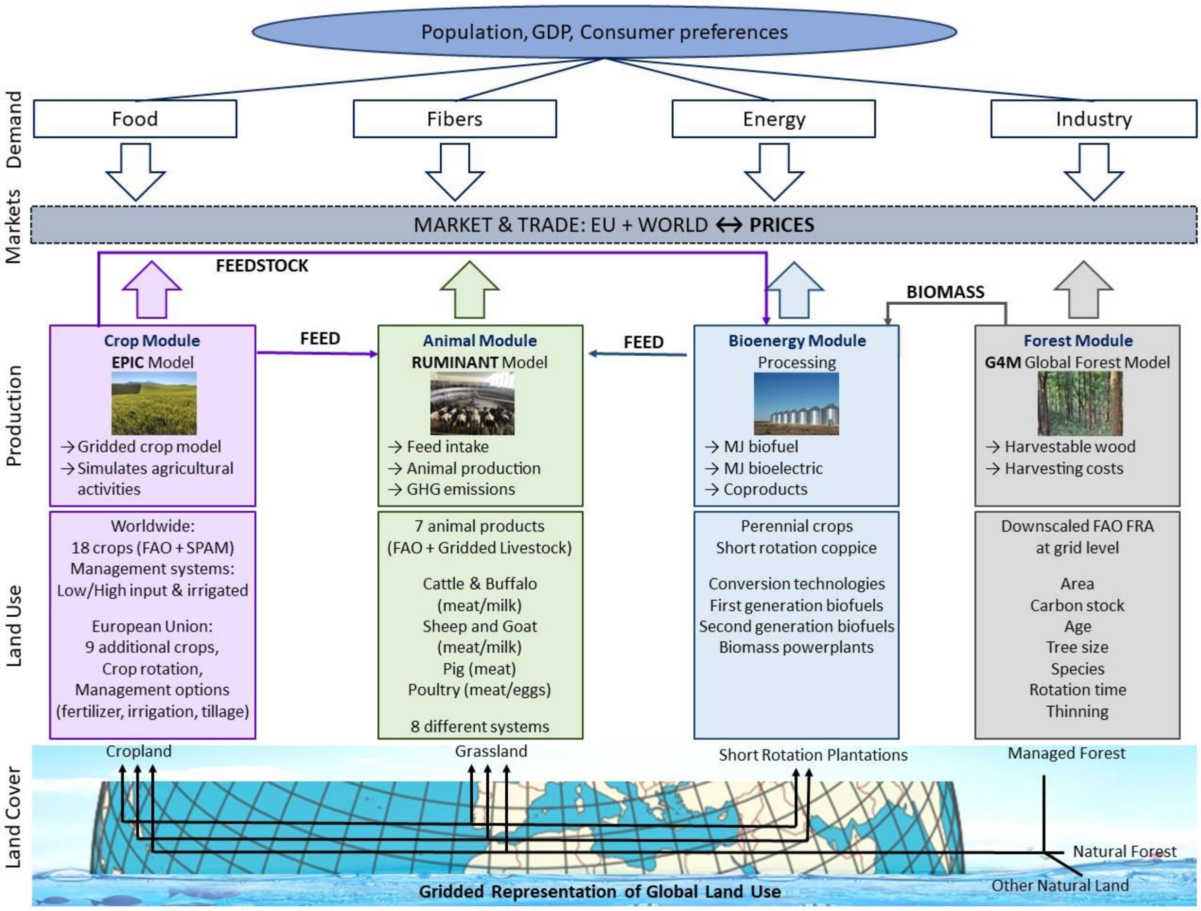

Figure S9 Schematic presentation of the GLOBIOM model

Food demand projections are based on the interaction of three different drivers: population growth, income per capita growth, response to prices. Population growth and income per capita growth are exogenously introduced in the model baseline. Demand increases linearly with population in each of the GLOBIOM regions. GDP per capita changes determine demand variation depending on income elasticity values associated to each scenario. Price effect is endogenously computed, and the final demand in the model is therefore influence by some other assumptions on technology, natural resources, etc. that shape price patterns.

GLOBIOM projects the under-nourished population[1]. It is a multiple of the prevalence of undernourishment and the total population. Following the FAO methodology, the prevalence of under-nourishment is calculated using three key factors: the mean dietary energy availability (kcal per person per day), the mean minimum dietary energy requirement (MDER) and the coefficient of variation of the domestic distribution of dietary energy availability in a country. The food distribution in a country is assumed to obey a log-normal distribution, which is determined by the mean food calorie availability (mean) and the equity of the food distribution (variance). The proportion of the population under the cut- off point (MDER) is then defined as the prevalence of under-nourishment. The calorie-based food consumption (kcal per person per day) output from the model is used for the mean food calorie availability. The future mean MDER is calculated for each year and country using the mean MDER in the base year at the country, adjusted for the MDER in different age and sex groups and future population demographics to reflect differences in the MDER across age and sex. The future equity of food distribution is estimated by applying the historical trend of income growth and the improved coefficient of variation of the food distribution to the future, such that the equity is improved along

with income growth in future at historical rates up to the present best value (0.2). No risk of hunger for high-income countries where hunger is not currently reported is assumed.

GLOBIOM covers major greenhouse gas (GHG) emissions from Agriculture, Forestry and Other Land Use (AFOLU) based on IPCC accounting guidelines including N2O from application of synthetic fertilizer and manure to soils, N2O from manure dropped on pastures, CH4 from rice cultivation, N2O and CH4 from manure management, and CH4 from enteric fermentation, and CO2 emissions/removals from above- and below-ground biomass changes for other natural vegetation. CO2 emissions/removals from afforestation, deforestation, wood production in managed forests are estimated by geographically explicit (0.5x0.5 degree) model Global Forest Model (G4M)[2,3] that is connected with GLOBIOM.

In addition, GLOBIOM endogenously represents a comprehensive set of mitigation technologies for the crop- and livestock sectors including technological options (based on the US EPA dataset of non-CO2 abatement technologies[4]), structural options (e.g. transition in production systems, changes in regional product mix, international trade), and demand side (alternative diets, consumer response to price signals) mitigation options.

Commodity markets and international trade are modelled at the level of various aggregate economic regions (that can change depending on user needs) where prices are endogenously determined at the regional level to establish market equilibrium. Trade is modelled following the spatial equilibrium approach based on cost competitiveness and homogeneous good assumption that allows for tracing of bilateral trade flows between individual regions. Besides primary products for the different sectors, the model has several final and by-products, for which, processing activities are defined.

GLOBIOM runs recursively from the base year to a future time horizon (often 2030, 2050 or 2100), and is designed to explore future scenarios. Future scenarios can be implemented through updated model parameters (e.g., population, GDP, crop yields, etc.) as well as new parameters and equations (e.g., introduction of a tax or constraints on the values of specific model variables, introduction of a new supply chain). GLOBIOM is run following recursive dynamics. Contrary to fully dynamic models, the agents of the economy do not make strategic decisions taking into account the future value of some parameters over several periods of time. However, the optimal decision in time period t depends on some decisions that agents have taken in the previous time period t-1. For instance, in GLOBIOM, at the beginning of the next period, the starting conditions for land use are updated using the solutions of the simulations from the previous period. Moreover, the reference is updated for each time step using exogenous drivers. For crops, livestock products and timber products, projections of population and GDP growth per region[5] are used to set up the initial demand level before market adjustments.

References

1. Hasegawa, T., Havlík, P., Frank, S., Palazzo, A. & Valin, H. Tackling food consumption inequality to fight hunger without pressuring the environment. *Nat. Sustain.* **2**, 826–833 (2019).

2. Gusti, M. *et al.* The sensitivity of the costs of reducing emissions from deforestation and degradation (REDD) to future socioeconomic drivers and its implications for mitigation policy design. *Mitig. Adapt. Strateg. Glob. Chang.* **24**, 1123–1141 (2019).

3. Gusti, M. & Kindermann, G. An approach to modeling landuse change and forest management on a global scale. in *1st International Conference on Simulation and Modeling Methodologies, Technologies and Applications (SIMULTECH 2011)* (2011).

4. Beach, R. H. *et al.* Global mitigation potential and costs of reducing agricultural non-CO2

greenhouse gas emissions through 2030. *J. Integr. Environ. Sci.* **12**, 87–105 (2015).

5. Nakicenovic, N. & Swart, R. *Emissions scenarios - special report of the Intergovernmental Panel on Climate Change*. (2000).
