# Supplementary material for: Feeding climate and biodiversity goals with novel plant-based meat and milk alternatives
Source: Nat Commun. 2023 Sep 12;14:5316. doi: 10.1038/s41467-023-40899-2 (PMC10497520; doi:10.1038/s41467-023-40899-2)
Supplement: Supplementary file 3 — Reporting Summary [file 41467_2023_40899_MOESM3_ESM.pdf]

nature portfolio

Corresponding author(s): Marta Kozicka

Last updated by author(s): Jul 24, 2023

Reporting Summary

Nature Portfolio wishes to improve the reproducibility of the work that we publish. This form provides structure for consistency and transparency in reporting. For further information on Nature Portfolio policies, see our Editorial Policies and the Editorial Policy Checklist.

Statistics

For all statistical analyses, confirm that the following items are present in the figure legend, table legend, main text, or Methods section.

n/a	Confirmed	
☒	☐	The exact sample size (*n*) for each experimental group/condition, given as a discrete number and unit of measurement
☒	☐	A statement on whether measurements were taken from distinct samples or whether the same sample was measured repeatedly
☒	☐	The statistical test(s) used AND whether they are one- or two-sided<br>*Only common tests should be described solely by name; describe more complex techniques in the Methods section.*
☒	☐	A description of all covariates tested
☒	☐	A description of any assumptions or corrections, such as tests of normality and adjustment for multiple comparisons
☒	☐	A full description of the statistical parameters including central tendency (e.g. means) or other basic estimates (e.g. regression coefficient) AND variation (e.g. standard deviation) or associated estimates of uncertainty (e.g. confidence intervals)
☒	☐	For null hypothesis testing, the test statistic (e.g. *F*, *t*, *r*) with confidence intervals, effect sizes, degrees of freedom and *P* value noted<br>*Give P values as exact values whenever suitable.*
☒	☐	For Bayesian analysis, information on the choice of priors and Markov chain Monte Carlo settings
☒	☐	For hierarchical and complex designs, identification of the appropriate level for tests and full reporting of outcomes
☒	☐	Estimates of effect sizes (e.g. Cohen's *d*, Pearson's *r*), indicating how they were calculated

Our web collection on statistics for biologists contains articles on many of the points above.

Software and code

Policy information about availability of computer code

Data collection	No software was used
Data analysis	All analysis was performed in R 4.1 and GAMS 32.2<br>The Global Biosphere Management Model (GLOBIOM) documentation, links to GLOBIOM resources, GAMS script descriptions and dependency links that match the Trunk version of the GLOBIOM model are provided in a GitHub repository https://iiasa.github.io/GLOBIOM/introduction.html.<br>All R code used for data analysis and creating the figures, together with the list of R packages used, are provided together with the model output data in the Zenodo database under accession code https://doi.org/10.5281/zenodo.8169317.

For manuscripts utilizing custom algorithms or software that are central to the research but not yet described in published literature, software must be made available to editors and reviewers. We strongly encourage code deposition in a community repository (e.g. GitHub). See the Nature Portfolio guidelines for submitting code & software for further information.

Data

Policy information about availability of data

All manuscripts must include a data availability statement. This statement should provide the following information, where applicable:

- Accession codes, unique identifiers, or web links for publicly available datasets
- A description of any restrictions on data availability
- For clinical datasets or third party data, please ensure that the statement adheres to our policy

The data that support the findings of this study are from publicly available datasets, such as USDA and FAOStat. The main data-sources include:
1. Heuzé V., Tran G., Hassoun P., Bastianelli D., Lebas F., 2019. Cottonseed meal. Feedipedia, a programme by INRAE, CIRAD, AFZ and FAO. https://www.feedipedia.org/node/550 Last updated on February 8, 2019, 14:21
2. Heuzé V., Tran G., Archimède H., Renaudeau D., Lessire M., Lebas F., 2015. Sugarcane juice. Feedipedia, a programme by INRAE, CIRAD, AFZ and FAO. https://feedipedia.org/node/560 Last updated on October 8, 2015, 18:31
3. Heuzé V., Tran G., Sauvant D., Lebas F., 2015. Oil palm fronds and oil palm crop residues. Feedipedia, a programme by INRAE, CIRAD, AFZ and FAO. https://www.feedipedia.org/node/6916 Last updated on June 25, 2015, 11:18
4. Berk, Z. Technology of Production of Edible Flours and Protein Products from Soybeans. Technology of production of edible flours and protein products from soybeans vol. 97 http://www.fao.org/docrep/t0532e/t0532e00.htm#con (1992).
5. Östbring, K., Nilsson, K., Ahlström, C., Fridolfsson, A. & Rayner, M. Emulsifying and anti-oxidative properties of proteins extracted from industrially cold-pressed rapeseed press-cake. Foods 9, (2020).
6. USDA. FoodData Central. 2022 https://fdc.nal.usda.gov/
7. The database of the PREDICTS (Projecting Responses of Ecological Diversity In Changing Terrestrial Systems) project https://doi.org/10.1002/ece3.2579
8. https://www.fao.org/faostat/en/#data/FBS

The GLOBIOM output data generated in this study have been deposited in the Zenodo database under accession code https://doi.org/10.5281/zenodo.8169317

Human research participants

Policy information about studies involving human research participants and Sex and Gender in Research.

Reporting on sex and gender	N/A
Population characteristics	N/A
Recruitment	N/A
Ethics oversight	N/A

Note that full information on the approval of the study protocol must also be provided in the manuscript.

Field-specific reporting

Please select the one below that is the best fit for your research. If you are not sure, read the appropriate sections before making your selection.

☐ Life sciences ☐ Behavioural & social sciences ☒ Ecological, evolutionary & environmental sciences

For a reference copy of the document with all sections, see nature.com/documents/nr-reporting-summary-flat.pdf

Ecological, evolutionary & environmental sciences study design

All studies must disclose on these points even when the disclosure is negative.

Study description	We apply the Global Biosphere Management Model (GLOBIOM), a partial equilibrium bio-economic model to assess environmental, including climate and biodiversity, and food security impacts of a partial dietary shift away from the animal sourced foods to plant-based diets. The novel alternatives recipes were constructed using processed ingredients such as flours and protein concentrates.
Research sample	No population sample was taken. GLOBIOM uses a wide variety of data from numerous publicly available sources. To convert the recipe ingredients into the corresponding primary crop equivalents we used literature review and open data sources (see methods and supplementary materials for the full list).
Sampling strategy	No new data was collected, no sample size calculation was performed.
Data collection	No primary data was collected. Data on the processing conversions and yields of processed products and by-products was collected via literature review or downloaded from the open source datasets by the authors.

Timing and spatial scale	No new data was collected, above-mentioned datasets were collected in 2020.
Data exclusions	No data were excluded.
Reproducibility	The input data, code and software versions of the GLOBIOM model runs is stored; the model output data, code and software used for the analysis have been deposited in the Zenodo database under accession code https://doi.org/10.5281/zenodo.8169317.
Randomization	Not relevant.
Blinding	Not relevant.

Did the study involve field work? ☐ Yes ☒ No

Reporting for specific materials, systems and methods

We require information from authors about some types of materials, experimental systems and methods used in many studies. Here, indicate whether each material, system or method listed is relevant to your study. If you are not sure if a list item applies to your research, read the appropriate section before selecting a response.

Materials & experimental systems

n/a	Involved in the study
☒	☐ Antibodies
☒	☐ Eukaryotic cell lines
☒	☐ Palaeontology and archaeology
☒	☐ Animals and other organisms
☒	☐ Clinical data
☒	☐ Dual use research of concern

Methods

n/a	Involved in the study
☒	☐ ChIP-seq
☒	☐ Flow cytometry
☒	☐ MRI-based neuroimaging
